# Supplementary material for: Comparative genomics provides new insights into the diversity, physiology, and sexuality of the only industrially exploited tremellomycete: Phaffia rhodozyma
Source: BMC Genomics. 2016 Nov 9;17:901. doi: 10.1186/s12864-016-3244-7 (PMC5103461; doi:10.1186/s12864-016-3244-7)
Supplement: Additional file 6: — List of orphan genes with links to PFAM (related to Additional file 1: Table S1). (ZIP 1428 kb) [file 12864_2016_3244_MOESM6_ESM.zip › PFAM_UNIQUE/G00619_P.html]

#                                                                            --- full sequence --- -------------- this domain -------------   hmm coord   ali coord   env coord
  
# target name        accession   tlen query name           accession   qlen   E-value  score  bias   #  of  c-Evalue  i-Evalue  score  bias  from    to  from    to  from    to  acc description of target
  
#------------------- ---------- ----- -------------------- ---------- ----- --------- ------ ----- --- --- --------- --------- ------ ----- ----- ----- ----- ----- ----- ----- ---- ---------------------
  
G00619\_P             -            692 DUF1421              PF07223.6    358     0.026   13.0   0.1   1   2       4.3   1.1e+03   -2.3   8.4    33   133   143   254   120   306 0.49 - Protein of unknown function (DUF1421)  
G00619\_P             -            692 DUF1421              PF07223.6    358     0.026   13.0   0.1   2   2    0.0001     0.026   13.0   0.1    41   173   433   637   413   655 0.70 - Protein of unknown function (DUF1421)
